# Inflammation as a risk factor for the development of frailty in the Lothian Birth Cohort 1936

**DOI:** 10.1016/j.exger.2020.111055

**Published:** 2020-10-01

**Authors:** Miles Welstead, Graciela Muniz-Terrera, Tom C. Russ, Janie Corley, Adele M. Taylor, Catharine R. Gale, Michelle Luciano

**Affiliations:** a Lothian Birth Cohorts, School of Philosophy, Psychology & Language Sciences, 7 George Square, University of Edinburgh, Edinburgh, UK; b Edinburgh Dementia Prevention, University of Edinburgh, BioCube 1, Edinburgh, UK; c Alzheimer Scotland Dementia Research Centre, 7 George Square, University of Edinburgh, Edinburgh, UK; d MRC Lifecourse Epidemiology Unit, University of Southampton, Southampton, UK

**Keywords:** Trajectory, Risk factor, Healthy ageing, Longitudinal

## Abstract

**Background:**

Research suggests that frailty is associated with higher inflammation levels. We investigated the longitudinal association between chronic inflammation and frailty progression.

**Methods:**

Participants of the Lothian Birth Cohort 1936, aged 70 at baseline were tested four times over 12 years (wave 1: *n* = 1091, wave 4: *n* = 550). Frailty was assessed by; the Frailty Index at waves 1–4 and Fried phenotype at waves 1, 3 and 4. Two blood-based inflammatory biomarkers were measured at wave 1: Fibrinogen and C-reactive protein (CRP).

**Results:**

Fully-adjusted, linear mixed effects models showed higher Fibrinogen was significantly associated with higher wave 1 Frailty Index score (β = 0.011, 95% CI[0.002,0.020], *p* < .05). Over 12 year follow-up, higher wave 1 CRP (β = 0.001, 95% CI[0.000,0.002], *p* < .05) and Fibrinogen (β = 0.004, 95% CI[0.001,0.007], *p* < .05) were significantly associated with increased Frailty Index change. For the Fried phenotype, wave 1 Pre-frail and Frail participants had higher CRP and Fibrinogen than Non-frail participants (*p* < .001). Logistic regression models calculated risk of worsening frailty over follow-up and we observed no significant association of CRP or Fibrinogen in minimally-adjusted nor fully-adjusted models.

**Conclusions:**

Findings showed a longitudinal association of higher wave 1 CRP and Fibrinogen on worsening frailty in the Frailty Index, but not Fried Phenotype. A possible explanation for this disparity may lie in the conceptual differences between frailty measures (a biopsychosocial vs physical approach). Future research, which further explores different domains of frailty, as well the associations between improving frailty and inflammation levels, may elucidate the pathway through which inflammation influences frailty progression. This may improve earlier identification of those at high frailty risk.

## Highlights

• Research exploring inflammation and frailty change over time is lacking.
• Inflammation is associated with frailty over time when measured by a Frailty Index.
• Understanding this association may help improve frailty interventions.
• Results differ according to the tool used to measure frailty.
• Future research should continue to compare different ways of measuring frailty.

## 1 Introduction

Although a definitive definition of frailty has yet to be established, it is generally accepted to refer to a clinical syndrome associated with an increased state of vulnerability in older adults (Iwasaki et al., 2018). This vulnerability increases an individual's risk of injury, disability, hospitalisation, and mortality (Fried et al., 2001). However, our understanding of frailty's aetiology remains poor (Hubbard and Woodhouse, 2010). Inflammation is a defence response undertaken by the immune system to combat harmful factors affecting the body (Pawelec et al., 2014). However, particularly in later life, chronic inflammation at low levels (inflamm-ageing) may develop even in the absence of infection (Samson et al., 2019). In order to measure inflammation, markers obtained from blood samples are often used. For instance, Fibrinogen, a plasma protein synthesised in the liver increases in the blood in response to systemic inflammation (Davalos and Akassoglou, 2012). Similarly, C-reactive protein (CRP), an acute phase protein found in blood plasma increases in concentration in response to inflammatory cytokines like Interleukin 6 (IL-6), and thus acts as a reliable indicator of inflammation in the body (Sproston and Ashworth, 2018). Elevated levels of inflammatory markers like Fibrinogen and CRP are consistently found amongst older people (Singh and Newman, 2011) and could potentially contribute to an increased risk of various diseases in later life (Pawelec et al., 2014; Sanada et al., 2018). Much like frailty, inflamm-ageing is seen as a significant risk factor for morbidity and mortality (Sanada et al., 2018) and is more pronounced in women (Samson et al., 2019). A recent systematic review and meta-analysis of 31 cross-sectional studies showed that frail and pre-frail individuals had significantly higher levels of inflammatory markers, including Fibrinogen and CRP (Soysal et al., 2016, Soysal et al., 2017). These findings make the interaction between frailty and inflammation of particular interest.

A salient issue in frailty research is the surplus of measurement tools available. Frailty is measured differently both in conception and operationalisation. Two of the main measurement tools illustrate this disparity: the Fried phenotype measures frailty according to five physical measurements (weight loss, exhaustion, level of physical activity, walking speed, and weakness) and categorises individuals as Non-frail, Pre-frail, or Frail (Fried et al., 2001); the Frailty Index (FI) measures frailty as a continuous variable according to at least 30 physical, psychological, and social deficits across an individual's life (Mitnitski et al., 2001; Rockwood and Mitnitski, 2007). As far as we are aware, only four publications have examined the longitudinal association between inflammation and frailty, three of which used the Fried phenotype (Reiner et al., 2009; Baylis et al., 2013; Gale et al., 2013), and one (Puts et al., 2005) which used a self-created but unvalidated measure based on nine physical and psychological frailty indicators. In a meta-analysis of the four studies, no overall association was observed between inflammatory markers and incidence of frailty over time (Soysal et al., 2016, Soysal et al., 2017). In a 2005 frailty and inflammation paper, the lack of longitudinal research exploring this association was discussed (Puts et al., 2005). Over a decade later a 2016 review paper highlighted that there remains a need for more of this research (Soysal et al., 2016, Soysal et al., 2017).

Here we test the association between frailty and inflammation in the Lothian Birth Cohort 1936 (LBC1936). By exploring the association of inflammation and frailty over time, it may be possible to determine markers which are able to predict frailty risk. This could have important implications for public health intervention strategies for the care of elderly people. A recent systematic review (Welstead et al., 2020), concluded that, in lieu of a gold standard frailty measurement tool, it may be beneficial to utilise multiple measures. Subsequently, we used both the FI and the Fried phenotype to assess associations with inflammation and evaluate any potential differences in findings according to the measure used. To our knowledge, no previous longitudinal studies have explored frailty and inflammation in this manner. Our goal was to test the association between baseline inflammation levels and progression of frailty by end of follow-up, 12 years later. We hypothesised that those with higher baseline inflammation levels would also have an increased level of baseline frailty in both frailty measures. Over the follow-up, we predicted that higher baseline inflammation would be associated with a steeper trajectory of FI change during follow-up and a higher risk of Fried phenotype transition from Non-Frail to Pre-Frail or Frail.

## 2 Methods

### 2.1 Study sample

From 2004 to 2007, 1091 participants from the Lothian Birth Cohort 1936 (LBC1936) with a mean (SD) age of 69 (0.83) years, 49.8% female, were recruited and tested at baseline. Follow-up waves were conducted every three years spanning 12 years in total (wave 2 *n* = 866, wave 3 *n* = 697, wave 4 *n* = 550). Sample attrition across follow-up left 550 participants at wave 4. Table 1 reports summary information at each wave. For more details on the cohort, see the LBC1936 profile papers (Deary et al., 2011; Taylor et al., 2018; Deary et al., 2007). LBC1936 was conducted according to the Declaration of Helsinki guidelines with ethical permission obtained from the Multi-Centre Research Ethics Committee for Scotland (MREC/01/0/56), Lothian Research Ethics Committee (LREC/2003/2/29), and Scotland A Research Ethics Committee (07/MRE00/58). Written consent was obtained from all participants.

**Table 1:** Summary characteristics of participants at each LBC1936 wave.

Variables	Wave 1 (Baseline)	Wave 2	Wave 3	Wave 4
Participants (*n*)	1091	866	697	550
Age in years, mean (SD)	69.6 (0.8)	72.5 (0.7)	76.3 (0.7)	79.4 (0.6)
Female, *n* (%)	543 (49.8%)	418 (48.3%)	337 (48.4%)	275 (50%)
FI, mean (SD)	0.16 (0.1)	0.18 (0.1)	0.20 (0.1)	0.21 (0.1)
Fried phenotype, *n* (%) Non-frail Pre-frail Frail	478 (44%) 520 (48%) 93 (8%)	Insufficient data to construct phenotype	269 (39%) 326 (47%) 102 (14%)	222 (40%) 259 (47%) 69 (13%)
CRP (mg/l), mean (SD)	3.5 (2.4)	3.3 (2.4)	2.5 (2.3)	2.4 (2.2)
Fibrinogen (g/L), mean (SD)	3.3 (0.6)	3.3 (0.6)	3.0 (0.6)	3.1 (0.5)

Note. CRP: C - reactive protein; FI: Frailty Index.

### 2.2 Inflammation measures

At baseline, blood samples were drawn, and of interest to this study, analysed for two commonly used biomarkers of inflammation: CRP (mg/l) and Fibrinogen (g/L) (Del Giudice and Gangestad, 2018). CRP assays were undertaken with a dry slide immune-rate method with an OrthoFusion 5.1 FS analyser. Consistent with previous research (Corley et al., 2015), CRP values over 10 mg/l were excluded from analysis due to the likelihood that they represent acute illness. CRP distributions were positively skewed, however none of the transformations tried improved this distribution, and for the sake of interpretability, measures were left untransformed. Furthermore, inspection of residuals did not identify departure from distributional assumptions. Fibrinogen samples were obtained with a Clauss assay (Luciano et al., 2009), and measures were normally distributed and no values were excluded.

### 2.3 Frailty measures

The FI was constructed at each wave according to pre-established guidelines (Searle et al., 2008). We included 30 deficits covering different body systems (psychological, cognitive, and physical). Whilst some cut-off values were clear (e.g. a disease is present or absent), others were not (e.g. grip strength), in these cases previously established methods were used (Searle et al., 2008). Deficits and cut-off values are reported in Table A1. For each participant the number of present deficits was summed and divided by the total number of deficits (*n* = 30). Computed scores ranged from 0 to 1, with higher scores representing a higher degree of frailty.

The Fried phenotype is based on five pre-specified dimensions: weight loss, exhaustion, physical health, walking speed, and grip strength. The presence of one or two of these dimensions indicated that an individual is Pre-frail, whilst three or more indicated Frailty. Fried phenotype was calculated at all waves other than wave 2 due to insufficient data. Full details are reported in Appendix A.

### 2.4 Covariates

For FI and Fried phenotype analyses we included covariates: age, sex, smoking status (current/ex/never), alcohol intake (units per week), years of formal full-time education, occupational social class (professional/managerial/skilled, non-manual/skilled manual or semiskilled/unskilled), and childhood IQ (measured with the Moray House Test in the LBC1936 at age 11) (Penrose, 1949). Childhood IQ was included as a covariate due to previous findings in the LBC1936 indicating that lower intelligence in childhood is associated with increased inflammation (Luciano et al., 2009) and an increased risk of frailty in older age (Gale et al., 2016). For details on how social class and Childhood IQ was derived, see Appendix B. Additionally, for Fried phenotype analyses we added covariates that were not included for FI analyses due to their inclusion in the composition of the measure. These included: self-reported history of various chronic diseases, depressive symptoms from the Hospital Anxiety and Depression scale (HADS) (Zigmond and Snaith, 1983) and Body Mass Index (BMI). As one of the HADS questions was included in the composition of the Fried Phenotype, this question was removed when deriving the depressive symptoms covariate.

### 2.5 Missing data

Over the four waves, there were a small number of instances where it was not possible to take certain measures for some participants. In these instances we used multiple imputation with the MICE package in R version 3.5.3 (Buuren and Groothuis-Oudshoorn, 2010; R Core Team, 2018). Five imputations were used to estimate missing data needed for the creation of our frailty measures, and a total of 49 missing values were replaced with substituted values.

### 2.6 Statistical analyses

**Fig. 1:**
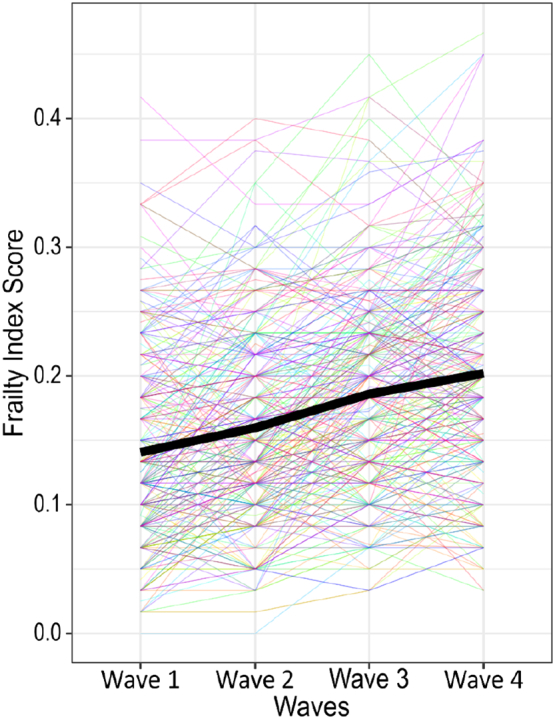
A plot of Frailty Index trajectories and estimated mean over the course of follow-up.

Due to the differences in how frailty is quantified in the Fried phenotype (categorical) and the FI (continuous), we used different statistical techniques for each measure. Linear mixed effects models using the LME4 package in R (R Core Team, 2018) were used to estimate change in FI scores from baseline to wave 4 and evaluate the association between baseline CRP and Fibrinogen and frailty trajectories. Models describing linear and accelerating change were fitted and adjusted for covariates, and then the best fitting model was chosen according to BIC fit indices. Fig. 1 illustrating the progression of FI over time was created using the GGPlot2 function in R (R Core Team, 2018).

Logistic regression was undertaken with the GLM function in R (R Core Team, 2018) to calculate the association between baseline CRP and Fibrinogen on the odds of frailty transition in the Fried phenotype between baseline and wave 4 (transition/no transition). Transitions were considered present if there was a worsening in frailty status i.e. from Non-Frail to Pre-Frail or Frail, or Pre-frail to Frail. Improvements in frailty status were also seen over the follow-up period with approximately 12% of participants showing an improvement in frailty status. Due to the focus of our study on frailty decline, these cases were not included in our analyses. Pearson's correlation coefficients were used to assess the inter-relationships between the inflammatory markers. An initial baseline model was calculated which controlled for age and sex, before computing a final model adjusting for other covariates. *t*-tests were used to describe sex group differences and assess baseline associations between inflammation and baseline frailty. Due to the use of two separate outcomes, there was no requirement for multiple testing corrections.

## 3 Results

**Fig. A1:**
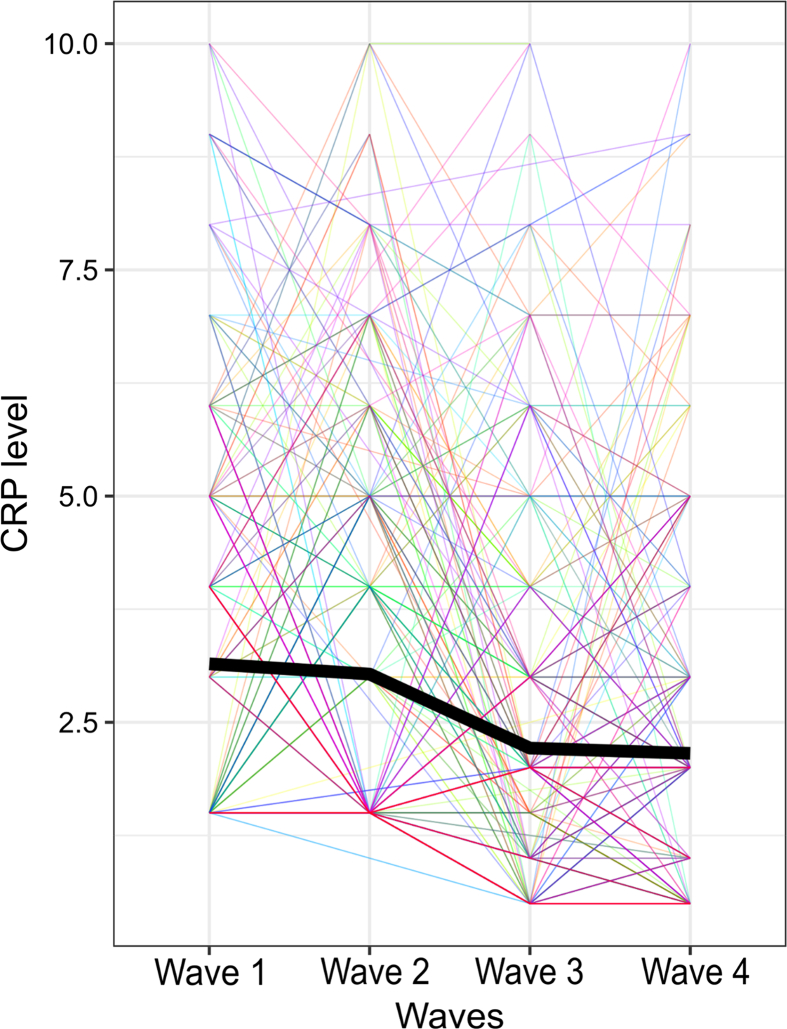
A plot of CRP levels the course of follow-up.

**Fig. A2:**
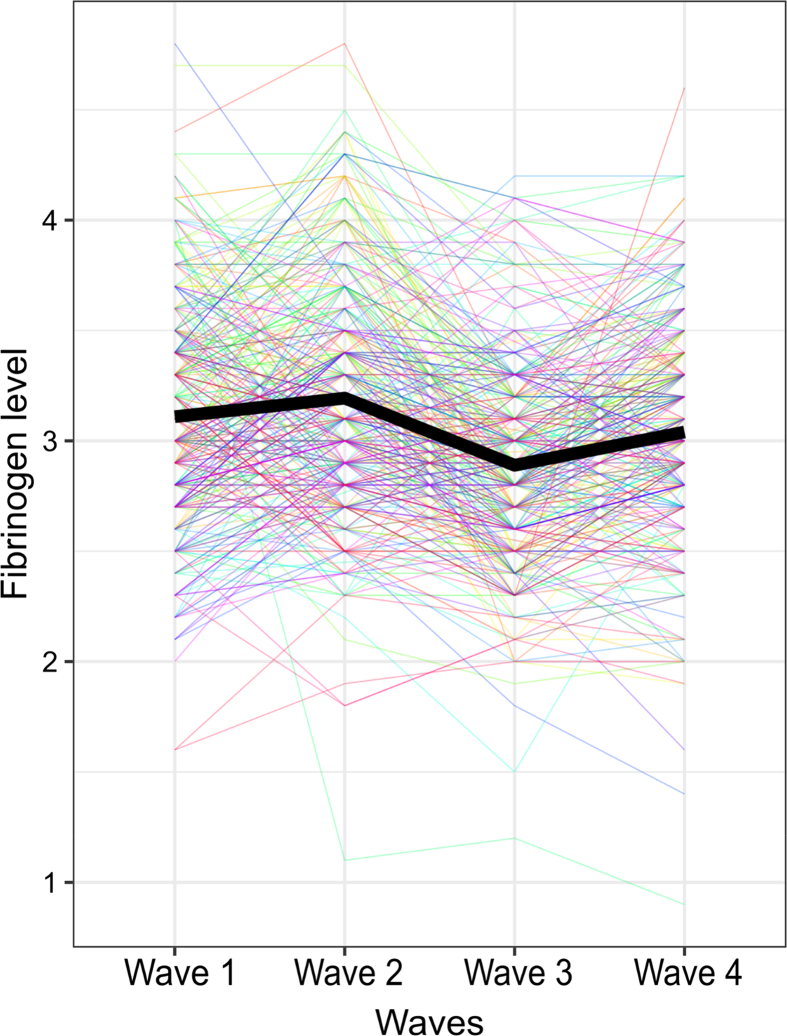
A plot of Fibrinogen levels over the course of follow-up.

At baseline, a moderate correlation was seen between the Fried phenotype and the FI (rho = 0.43). This relationship was consistent at waves 3 and 4, where both frailty measures were also available (rho = 0.51 & 0.48, respectively). Baseline CRP and Fibrinogen showed a low positive correlation (rho = 0.28). T-tests found significantly higher levels of baseline FI scores for those who withdrew from the study compared to those who completed all waves (completers mean [SD] = 0.15 [0.08], withdrawers mean [SD] = 0.18 [0.09]; t[1065] = 6.06, *p* < .001). A significant difference between Fried Phenotype category and completers vs withdrawers was also found (t[1080] = 4.43, *p* < .001). Findings showed that 11.3% of withdrawers compared to 5.8% of completers were categorised as frail by the Fried Phenotype at baseline. In total 145 out of a total 550 completers (26%) showed a transition to a worse frailty status over the follow-up period. Additionally, levels of baseline inflammation were higher for those who withdrew (CRP: completers mean [SD] = 3.27 [2.32], withdrawers mean [SD] = 3.69 [2.55]; t[901] = 2.60, *p* < .01, Fibrinogen: completers mean [SD] = 3.22 [0.59], withdrawers mean [SD] = 3.34 [0.68], t[1006] = 3.21, *p* < .01). Over the four waves of data, both CRP and Fibrinogen showed a small decrease, as seen in Fig. A1, Fig. A2.

### 3.1 Frailty index (FI)

At baseline, no significant sex difference in the FI was observed (male mean [SD] = 0.16 [0.08], female mean [SD] = 0.17 [0.09]; t[1088] = −1.35, *p* = .18). The comparison of fit indices between models describing the trajectory change in FI at a constant rate and models describing an accelerating rate of change showed that the best-fitting model was a model that considers FI change as constant and linear (CRP model BIC = −6826; Fibrinogen model BIC = −7646). Results of both CRP and Fibrinogen models indicated a significant association of time and FI scores, that is, scores increased on average by 0.030 (95% CI:[0.01, 0.05], *p* < .01,) with each wave. Fig. 1 shows this increase across waves. Random effects estimated the average variance of FI at baseline (SD = 0.07) and rate of FI change (SD = 0.02). Older age and lower childhood IQ were both associated with an increased baseline FI (*p* < .001). In the CRP model, baseline CRP did not have a significant association with baseline FI score but did show a significant association with the slope of FI change longitudinally (β = 0.001, 95% CI: [0.000, 0.002], *p* < .05). In the Fibrinogen model, baseline Fibrinogen was shown to have a significant association with baseline FI score (β =0.011, 95% CI: [0.002, 0.020], *p* < .05) as well as a significant association with the slope of FI change longitudinally (β =0.004, 95% CI: [0.001, 0.007], *p* < .05). Full results are reported in Table 2.

**Table 2:** Results from the linear mixed effects models assessing Frailty Index change in the LBC1936.

	CRP (mg/l) linear mixed effects model (BIC = −6826)			Fibrinogen (g/L) linear mixed effects model (BIC = −7646)		
Fixed effects	β	95% CI	p-Value	β	95% CI	p-Value
Rate of change	0.031	0.008, 0.052	0.006⁎⁎	0.029	0.008, 0.050	0.007⁎⁎
Inflammation	0.002	−0.000, 0.004	0.113	0.011	0.002, 0.020	0.016⁎
Age	0.000	0.000, 0.000	0.000⁎⁎⁎	0.000	0.000, 0.000	0.000⁎⁎⁎
Sex	0.012	−0.000, 0.025	0.055	0.013	0.001, 0.025	0.038⁎
Smoking	0.010	−0.002, 0.022	0.114	0.010	−0.001, 0.022	0.085
Alcohol intake	0.000	−0.000, 0.001	0.800	−0.000	−0.000, 0.000	0.949
Social class	−0.003	−0.009, 0.003	0.343	−0.005	−0.011, 0.001	0.117
Childhood IQ	−0.001	−0.002, −0.001	0.000⁎⁎⁎	−0.001	−0.002, −0.000	0.000⁎⁎⁎
Years of education	−0.004	−0.011, 0.002	0.160	−0.005	−0.011, 0.000	0.096
Inflammation over time	0.001	0.000, 0.002	0.021⁎	0.004	0.001, 0.007	0.014⁎
Age over time	−0.000	−0.000, 0.000	0.115	−0.000	−0.000, 0.000	0.410
Sex over time	−0.003	−0.007, 0.001	0.193	−0.004	−0.008, 0.000	0.092
Smoking over time	0.004	−0.000, 0.008	0.055	0.005	0.001, 0.009	0.009⁎⁎
Alcohol intake over time	−0.000	−0.000, 0.000	0.247	−0.000	−0.000, 0.000	0.385
Social class over time	−0.001	−0.003, 0.001	0.311	−0.001	−0.001, 0.009	0.524
Childhood IQ over time	−0.000	−0.000, 0.000	0.538	−0.000	−0.000, 0.000	0.500
Years of education over time	0.001	−0.002, 0.003	0.583	0.000	−0.002, 0.002	0.702

Note. CRP: C - reactive protein; FI: Frailty Index.

Units: Inflammation: CRP or Fibrinogen respectively; Smoking: Current, Ex, Never; Alcohol intake: units per week; Social class: professional, managerial, skilled non-manual, skilled manual, and semiskilled/unskilled.

⁎⁎⁎ *P* < .001.

⁎⁎ *P* < .01.

⁎ *P* < .05.

### 3.2 Fried phenotype

At baseline there was a significant difference between Fried phenotype category membership and CRP (*p* < .001) and Fibrinogen (*p* < .001). Non-Frail participants had lower CRP (mean [SD] = 3.16 [2.26]) and Fibrinogen (mean [SD] = 3.17 [0.55]) than Pre-Frail participants (CRP mean [SD] = 3.68 [2.55], Fibrinogen mean [SD] = 3.32 [0.65]) or Frail participants (CRP mean [SD] = 4.07 [2.58], Fibrinogen mean [SD] = 3.60 [0.82]). The distribution of men and women did not differ significantly by baseline Fried phenotype category. Further cross-sectional results showed significant differences between Fried Phenotype categories and several covariates. Those in the Frail category had lower childhood IQ (*p* < .001), less education (*p* < .001), higher BMI (*p* < .001), higher depressive symptoms (*p* < .001), and higher instances of various chronic diseases including diabetes (*p* < .001), cardiovascular disease (*p* < .001), high cholesterol (*p* < .01), stroke (*p* < .01), Parkinson's disease (*p* < .05), and arthritis (*p* < .001). Frail individuals were also more likely to identify as a current smoker (*p* < .01) and more likely to belong to a lower occupational social class (*p* < .001). Full details of baseline Fried Phenotype differences are reported in Table A2.

Longitudinally, sex did not emerge as associated with the rate of transition between Fried phenotype categories. Of the 550 participants who completed follow-up, 5.8% were classified as frail at baseline compared to 12.5% at wave 4. Logistic regression models were used independently for CRP and Fibrinogen. In the baseline models with age and sex as covariates, neither CRP nor Fibrinogen showed a significant association with frailty transitions. Results in the fully-adjusted models remained non-significant both inflammatory biomarkers. Furthermore, no covariates showed significant associations with frailty transition. Full details are reported in Table 3.

**Table 3:** Results from the fully-adjusted logistic regression models assessing risk of Fried phenotype transition in the LBC1936.

Variables	CRP (mg/l) logistic regression model (AIC = 548)		Fibrinogen (g/L) logistic regression (AIC = 612)	
	Odds ratios (95% CI)	p-Value	Odds ratios (95% CI)	p-Value
Inflammation marker	1.03 (0.93, 1.13)	0.55	0.84 (0.58, 1.20)	0.34
Age	1.21 (0.93, 1.58)	0.16	1.15 (0.89, 1.48)	0.27
Sex	1.01 (0.63, 1.63)	0.96	1.04 (0.66, 1.63)	0.86
Smoking Status	1.19 (0.76, 1.86)	0.44	1.30 (0.86, 1.98)	0.21
Alcohol intake	1.00 (0.98, 1.02)	0.88	1.00 (0.94, 1.02)	0.81
Years of Education	1.07 (0.86, 1.34)	0.52	1.09 (0.88, 1.33)	0.43
Social class	0.89 (0.64, 1.25)	0.51	0.91 (0.64, 1.24)	0.55
Childhood IQ	1.00 (0.98, 1.02)	0.89	1.00 (0.99, 1.08)	0.86
BMI	1.04 (0.98, 1.10)	0.15	1.03 (0.98, 1.08)	0.19
History of diabetes (Yes/No)	0.72 (0.25, 1.86)	0.52	1.24 (0.53, 2.76)	0.60
History of cardiovascular disease (Yes/No)	0.88 (0.50, 1.54)	0.67	0.78 (0.46, 1.31)	0.36
History of high cholesterol (Yes/No)	1.14 (0.69, 1.85)	0.61	1.23 (0.78, 1.93)	0.36
History of stroke (Yes/No)	0.93 (0.19, 3.51)	0.92	1.20 (0.38, 3.49)	0.74
History of thyroid disease (Yes/No)	1.22 (0.57, 2.47)	0.59	0.96 (0.46, 1.89)	0.90
History of cancer (Yes/No)	0.79 (0.35, 1.63)	0.54	0.90 (0.43, 1.77)	0.76
History of Parkinson's disease (Yes/No)	0.00 (N/A^a^)	0.98	0.00 (N/A^a^)	0.98
History of arthritis (Yes/No)	0.95 (0.61, 1.48)	0.83	0.96 (0.63, 1.45)	0.84
Number of depressive symptoms (HADS)	0.99 (0.88, 1.10)	0.83	0.99 (0.89, 1.10)	0.90

Note. BMI: Body Mass Index; CRP: C - reactive protein; FI: Frailty Index; HADS: Hospital and Anxiety Depression Scale.

*Units*: Inflammation marker: CRP, Fibrinogen; Smoking: Current, Ex, Never; Alcohol intake: units per week; Social class: professional, managerial, skilled non-manual, skilled manual, and semiskilled/unskilled.

a Unable to calculate 95% CI due to small sample of Parkinson's disease case.

## 4 Discussion

### 4.1 Summary of findings and comparison with other literature

In this study, we investigated the association between two baseline inflammatory markers CRP and Fibrinogen, and frailty, as measured by the FI and the Fried phenotype, over 12 years of follow-up. Our hypothesis that higher levels of baseline inflammation would be associated with higher baseline frailty scores was partially supported; Fibrinogen, but not CRP, was cross-sectionally associated with FI scores. Whilst for the Fried phenotype both inflammation markers were higher in Pre-Frail and Frail participants compared to Non-Frail, findings which are consistent with previous cross-sectional research (Soysal et al., 2016, Soysal et al., 2017). Our longitudinal findings showed no significant associations of inflammation factors and Fried phenotype transitions across the follow-up. These results support previous null findings reported in a meta-analysis of four longitudinal studies (Puts et al., 2005; Reiner et al., 2009; Baylis et al., 2013; Gale et al., 2013). However, we did find significant associations between both CRP and Fibrinogen on the FI slope of change, indicating that higher levels of these markers at baseline increase the gradient of FI score over time. This supports our hypothesis that rate of FI change is influenced by inflammation at baseline. Differences in findings between risk factors and these two frailty measures have been observed previously (Gale et al., 2018) and our findings that FI and Fried phenotype are only moderately correlated reinforces previous comparisons (Aguayo et al., 2017).

### 4.2 Interpretation

One possibility for the absence of a longitudinal association between inflammatory biomarkers and the Fried phenotype may be the general rates of healthiness in the LBC1936. As the LBC1936 is a self-selected volunteer sample from a relatively affluent area of Scotland, there are, on average, higher levels of healthiness when compared to the general population (Deary et al., 2007; Taylor et al., 2018). Thus, the greater restriction of range in our measures may underestimate the true size of effects in the general population. Furthermore, there was significant attrition which could have led to a healthy survivor effect whereby those who withdrew from the study were more likely to have had worsening frailty. This is congruent with findings that early withdrawers had higher levels of baseline frailty in both the FI and Fried phenotype. Previous analyses of the LBC1936 show that compared to those who stayed in the study, those who dropped out had significantly lower socioeconomic status, fitness levels, grip strength, and cognitive ability, all measures which could contribute to a higher level of Fried phenotype transition (Taylor et al., 2018). Additionally, although women had a marginally higher baseline FI score than men, this did not reach statistical significance. This result is incongruent with previous research which generally finds that women report higher FI levels than men (Gordon et al., 2017), and might reflect further the general healthiness of the LBC1936 (Deary et al., 2007; Taylor et al., 2018).

Another possible explanation for why CRP and Fibrinogen were associated with FI change but not Fried phenotype transitions is the substantial difference in their conceptualisation of frailty. Not only do the FI and Fried phenotype differ in the composition of their measures (biopsychosocial vs purely physical), it may also be that the scale differences (categorical vs continuous) add to our discrepant findings. Previous research has found similar differences, for example, Gale et al. (2018) utilised both the FI and Fried Phenotype to investigate social isolation and loneliness, finding different results depending on the frailty measure used. Aguayo et al. (2017) argued that different frailty scales are often based on different concepts of frailty and that they cannot be compared despite aiming to measure a similar outcome. Accordingly, it may be that inflammation does contribute to increased risk of frailty according to the FI's biopsychosocial definition of frailty but not the Fried Phenotype's physical definition. Further research is required to replicate these findings and tease out the differences between different types of frailty measurements and the associations of inflammatory biomarkers.

### 4.3 Implications for policy/care

Understanding the association between chronic inflammation and frailty progression may be useful for physicians targeting services for elderly people. For example, elevated inflammation may not indicate the need for immediate clinical care, however it may reinforce the benefit of lifestyle changes to potentially attenuate the risk of worsening frailty. The Fried phenotype, whilst unable to capture the subtle changes, may be more useful for detecting significant shifts in an individual's frailty status, indicating the requirement for immediate care and intervention. It may also be useful in an older population than the LBC1936 where frailty rates are higher and transitions are more substantial.

### 4.4 Strengths and limitations

A strength of this study is the use of different frailty measurement tools. Whilst the optimal way to measure frailty remains a matter of dispute it is important to consider that not all tools are consistent in their findings, and thus it is important to compare them before reaching firm conclusions. Future research may benefit from this method and reduce the heterogeneity in the field. This study also has limitations. Due to a lack of data at wave 2 we were unable to compute the Fried phenotype at all waves. Accordingly, we calculated transitions over a 12 year period whereby sample attrition took place. Future studies that are able to calculate transitions with less attrition may be able to draw more generalisable conclusions. Additionally, for our logistic regression models we only considered frailty transitions as those who recorded worsening frailty over time. We did not distinguish between those who either stayed healthy or showed improvement in frailty status over time. It may be the case that improvements in frailty are associated with reductions in inflammation. Future research may benefit from exploring this relationship further. A further limitation concerned our lack of inclusion of anti-inflammatory drugs as a covariate, which could have acted as a confounder on our results. Use of anti-inflammatory drugs typically increase in older age (Fowler et al., 2014) and this potentially explains the decreases in Fibrinogen and CRP over time as seen in Fig. A1, Fig. A2.

### 4.5 Conclusions

We sought to explore the association between inflammation and frailty change over time. As far as we are aware, we are the first study to explore the longitudinal association between inflammation and FI. We found differing results depending on the frailty measurement tool used; inflammation showed a significant association with frailty over time when measured by the FI but not the Fried phenotype. The differences in frailty conceptualisation (biopsychosocial vs solely physical) may underpin this difference and further research is required to fully understand these differences. The value of comparing different frailty measures has been shown here, and should be continued in future research so that a better understanding of how inflammatory marker associations vary between different frailty conceptualisations can be established. By doing so, it may be possible to facilitate policy and clinical care improvements whereby frailty risk can be identified early, via markers like inflammation, and effective interventions can be implemented.

## Funding

LBC1936 data collection and MW's PhD scholarship is funded by the Disconnected Mind project (funded by 10.13039/501100000629Age UK [MR/M01311/1] and 10.13039/501100000265MRC [G1001245/96099]).

## Availability of data and material

Data was obtained from the Lothian Birth Cohort 1936, more information can be found at https://www.lothianbirthcohort.ed.ac.uk/.

## Code availability

R script can be provided upon request.

## Declaration of competing interest

The authors have no competing interests to declare.

Age UK and MRC are involved in funding the recruitment and data collection for the Lothian Birth Cohort 1936. The sponsor had no role in the design, methods, analysis and preparation of paper.

## Figures and Tables

**Table A1:** Items constituting the Frailty Index and their coding/cut-off points in the LBC1936.

Items	Coding	Cut-offs based on
Systolic blood pressure	Bottom 5th percentile (1), 5th–20th percentile (0.5), Above 20th percentile (0)	Recommended technique where no established cut-offs available (Theou et al., 2015)
Diabetes (self-reported)	Yes (1) or No (0)	Already binary variable
High Cholesterol (self-reported)	Yes (1) or No (0)	Already binary variable
Heart problems (self-reported)	Yes (1) or No (0)	Already binary variable
Stroke or mini stroke (self-reported)	Yes (1) or No (0)	Already binary variable
Leg pain (self-reported)	Yes (1) or No (0)	Already binary variable
Blood circulation issues (self-reported)	Yes (1) or No (0)	Already binary variable
Thyroid Disorder (self-reported)	Yes (1) or No (0)	Already binary variable
Cancer (self-reported)	Yes (1) or No (0)	Already binary variable
Parkinson's disease (self-reported)	Yes (1) or No (0)	Already binary variable
Dementia (self-reported)	Yes (1) or No (0)	Already binary variable
Arthritis (self-reported)	Yes (1) or No (0)	Already binary variable
Any other chronic disease (self-reported)	Yes (1) or No (0)	Already binary variable
Polypharmacy (self-reported)	>4 medications (1), ≤4 medications (0)	Previous literature (Theou et al., 2013)
Body Mass Index (BMI)	18.5 to <25 (0), 25 to <30 (0.5), <18.5 or > equal to 30 (1)	Previous literature (Chamberlain et al., 2016)
6 m walk time (gait speed)	>10 s or physically unable (1), <10 s (0)	Previous literature (Hoogendijk et al., 2017)
Able to stand up from a chair	Yes (1) or No (0)	Already binary variable
Grip strength (strongest hand and stratified by sex and BMI)	Bottom 5th percentile (1), 5th–20th percentile (0.5), Above 20th percentile (0)	Recommended technique where no established cut-offs available (Theou et al., 2015)
Townsend Disability Scale (Townsend, 1979)	11–18 (1), 0–10 (0)	Previous literature (Matthews et al., 2016)
Peak Expiratory Flow rate (stratified by sex)	Bottom 5th percentile (1), 5th–20th percentile (0.5), Above 20th percentile (0)	Recommended technique where no established cut-offs available (Theou et al., 2015)
Forced expiratory volume (stratified by sex)	Bottom 5th percentile (1), 5th–20th percentile (0.5), Above 20th percentile (0)	Recommended technique where no established cut-offs available (Theou et al., 2015)
Depression (measured by the HADS) (Zigmond and Snaith, 1983)	11–21 (1), 8–10 (0.5), 0–7 (0)	Previous literature (Zigmond and Snaith, 1983)
Anxiety (measured by the HADS) (Zigmond and Snaith, 1983)	11–21 (1), 8–10 (0.5), 0–7 (0)	Previous literature (Zigmond and Snaith, 1983)
Mini-Mental State Examination (MMSE) (Folstein et al., 1975)	<10 (1), 11–17 (0.75), 18–20 (0.5), 20–24 (0.25), >24 (0)	Previous literature (Searle et al., 2008)
Digit Symbol(Wechsler, 2003)	Bottom 5th percentile (1), 5th–20th percentile (0.5), Above 20th percentile (0)	Recommended technique where no established cut-offs available (Theou et al., 2015)
Block Design(Wechsler, 2003)	Bottom 5th percentile (1), 5th–20th percentile (0.5), Above 20th percentile (0)	Recommended technique where no established cut-offs available (Theou et al., 2015)
Verbal Fluency(Wechsler, 2003)	Bottom 5th percentile (1), 5th–20th percentile (0.5), Above 20th percentile (0)	Recommended technique where no established cut-offs available (Theou et al., 2015)
Matrix Reasoning(Wechsler, 2003)	Bottom 5th percentile (1), 5th–20th percentile (0.5), Above 20th percentile (0)	Recommended technique where no established cut-offs available (Theou et al., 2015)
Reaction time test(Cox et al., 1993)	Bottom 5th percentile (1), 5th–20th percentile (0.5), Above 20th percentile (0)	Recommended technique where no established cut-offs available (Theou et al., 2015)
Delayed recall(Wechsler, 2003)	Bottom 5th percentile (1), 5th–20th percentile (0.5), Above 20th percentile (0)	Recommended technique where no established cut-offs available (Theou et al., 2015)

Note. HADS: Hospital Anxiety and Depression scale.

**Table A2:** Baseline differences in characteristics for each category of the Fried phenotype in the LBC1936.

Variables	Non-frail (*n* = 478)	Pre-frail (*n* = 520)	Frail (*n* = 93)	*p*-Value
CRP mg/L: mean (SD)	3.2 (2.3)	3.7 (2.6)	4.1 (2.6)	<0.001⁎⁎⁎
Fibrinogen g/L: mean (SD)	3.2 (0.6)	3.3 (0.7)	3.6 (0.8)	<0.001⁎⁎⁎
Sex, *n* (%) Male Female	245 (51%) 233 (49%)	259 (50%) 261 (50%)	44 (47%) 49 (53%)	0.8
Body mass index (BMI), mean (SD)	27.1 (3.7)	28.2 (4.5)	29.4 (5.8)	<0.001⁎⁎⁎
Smoking status, *n* (%) Current Ex Never	39 (8%) 203 (43%) 236 (49%)	66 (13%) 223 (43%) 231 (44%)	20 (21%) 39 (42%) 34 (37%)	<0.01⁎⁎
Alcohol (units per week), mean (SD)	11.4 (14.8)	10.2 (13.8)	7.94 (12.6)	0.1
Social class, *n* (%) Professional Managerial Skilled non-manual Skilled manual Semiskilled/Unskilled	104 (22%) 188 (40%) 104 (22%) 56 (12%) 18 (4%)	78 (15%) 185 (36%) 122 (24%) 108 (21%) 19 (4%)	8 (9%) 29 (33%) 20 (23%) 24 (27%) 7 (8%)	<0.001⁎⁎⁎
Childhood IQ, mean (SD)	102.1 (14.4)	98.9 (14.8)	95.2 (17.4)	<0.001⁎⁎⁎
Years of Education, mean (SD)	10.9 (1.2)	10.7 (1.1)	10.3 (0.9)	<0.001⁎⁎⁎
History of diabetes, *n* (%) Yes No	18 (4%) 460 (96%)	48 (9%) 472 (91%)	25 (27%) 68 (73%)	<0.001⁎⁎⁎
History of cardiovascular disease, *n* (%) Yes No	92 (19%) 386 (81%)	134 (26%) 386 (74%)	42 (45%) 51 (55%)	<0.001⁎⁎⁎
History of high cholesterol, *n* (%) Yes No	147 (31%) 330 (69%)	192 (37%) 328 (63%)	47 (51%) 46 (49%)	<0.01⁎⁎
History of stroke, *n* (%) Yes No	15 (3%) 463 (97%)	30 (6%) 490 (94%)	9 (10%) 84 (90%)	<0.05⁎
History of thyroid disease, *n* (%) Yes No	35 (7%) 443 (93%)	53 (10%) 467 (90%)	11 (12%) 81 (88%)	0.2
History of cancer, *n* (%) Yes No	61 (13%) 417 (87%)	58 (11%) 462 (89%)	15 (16%) 78 (84%)	0.4
History of Parkinson's disease, *n* (%) Yes No	2 (>1%) 476 (<99%)	1 (>1%) 519 (<99%)	2 (>1%) 91 (<99%)	<0.05⁎
History of arthritis, *n* (%) Yes No	175 (37%) 302 (63%)	241 (46%) 279 (54%)	61 (66%) 32 (34%)	<0.001⁎⁎⁎
Depressive symptoms, mean (SD)	1.24 (1.48)	1.69 (1.87)	3.53 (2.61)	<0.001⁎⁎⁎

⁎⁎⁎ *P* < .001.

⁎⁎ *P* < .01.

⁎ *P* < .05.

## Appendix A

### Deriving the fried criteria

The Fried Criteria was comprised on five dimensions. These were measured in the LBC1936 as follows;

#### Weight loss

Weight was measured using an electronic weighing scale, and height was measured in metres using a stadiometer. From this, it was possible to compute BMI by dividing weight by height squared. At baseline, weight loss was defined as a BMI less than 18.5 kg/m^2^. At waves 3 and 4, weight loss was defined as a loss of weight of 10% or more since their previous visit or a BMI less than 18.5 kg/m^2^.

#### Exhaustion

Exhaustion was measured using the Hospital Anxiety and Depression Scale (HADS) (Zigmond and Snaith, 1983). Exhaustion was scored as present if the participant responded ‘very often’ or ‘nearly all the time’ to the item ‘I feel as if I'm slowed down’.

#### Physical activity

A question asking participants about their usual level of physical activity was used with six responses ranging from moving only when necessary, to heavy exercise or sport several times a week. In line with previous publications (Gale et al., 2017), participants in the lowest sex-specific 20% of the distribution were defined as having low physical activity.

#### Walking speed

Participants were recorded walking a distance of six metres at maximum speed. After adjusting for sex and height, those in the lowest 20% of the distribution were considered to have a slow walking speed.

#### Weakness

Maximum grip strength was measured in all participants using a dynamometer. Participants were measured three times with the strongest attempt being used for analysis. After adjusting for sex and BMI, those in the lowest 20% of the distribution were defined as having weakness.

## Appendix B Defining occupational social class

Occupational social class was based upon principal occupation, coded in line with the 1980 census (General, 1991). Five social class categories were used: professional, managerial, skilled non-manual, skilled manual, and semiskilled/unskilled. The women in the cohort were asked for their husband's occupation as well as their own, and they were assigned a social class based on the highest occupation of the household. This was derived from their own occupation for about half of the women, and from their husband's occupation for the remainder.

Childhood IQ was derived from Moray House Test scores at age 11 (Penrose, 1949) as part of the LBC1936. Raw scores were corrected for age in days at time of testing and converted to an IQ scale where mean (SD) = 100 (15).

## Appendix C Supplementary data

Supplementary material Image 1
