# Supplementary material for: Inflammation as a risk factor for the development of frailty in the Lothian Birth Cohort 1936
Source: Exp Gerontol. 2020 Oct 1;139:111055. doi: 10.1016/j.exger.2020.111055 (PMC7456784; doi:10.1016/j.exger.2020.111055)
Supplement: Supplementary file 1 — Supplementary material [file mmc1.docx]

**Inflammation as a Risk Factor for the Development of Frailty in the Lothian Birth Cohort 1936**

Miles C. Welstead^a^ MSc*

Graciela Muniz-Terrera^b^ PhD

Tom C. Russ^a-c^ PhD MRCPsych

Janie Corley^a^ PhD

Adele Taylor^a^ MA

Catharine R. Gale^a-d^ PhD

Michelle Luciano^a^ PhD

^a^ Lothian Birth Cohorts, School of Philosophy, Psychology & Language Sciences, 7 George Square, University of Edinburgh, Edinburgh, UK;

^b^ Edinburgh Dementia Prevention, University of Edinburgh, BioCube 1, Edinburgh, UK;

^c^ Alzheimer Scotland Dementia Research Centre, 7 George Square, University of Edinburgh, Edinburgh, UK

^d^ MRC Lifecourse Epidemiology Unit, University of Southampton, Southampton, UK

***Corresponding author**

Miles Welstead, School of Philosophy, Psychology & Language Sciences, 7 George Square, University of Edinburgh, United Kingdom, EH8 9JZ

Email: [miles.welstead@ed.ac.uk](mailto:miles.welstead@ed.ac.uk)

ORCID: 0000-0003-4615-2539

**Online Resources**

**Online Resource 1: Items constituting the Frailty Index and their coding/cut-off points in the LBC1936**

| Items | Coding | Cut-offs based on |
| --- | --- | --- |
| Systolic Blood Pressure | Bottom 5^th^ percentile (1), 5^th^-20^th^ percentile (0.5), Above 20^th^ percentile (0) | Recommended technique where no established cut-offs available (Theou et al. 2015) |
| Diabetes (self-reported) | Yes (1) or No (0) | Already binary variable |
| High Cholesterol (self-reported) | Yes (1) or No (0) | Already binary variable |
| Heart problems (self-reported) | Yes (1) or No (0) | Already binary variable |
| Stroke or mini stroke (self-reported) | Yes (1) or No (0) | Already binary variable |
| Leg pain (self-reported) | Yes (1) or No (0) | Already binary variable |
| Blood circulation issues (self-reported) | Yes (1) or No (0) | Already binary variable |
| Thyroid Disorder (self-reported) | Yes (1) or No (0) | Already binary variable |
| Cancer (self-reported) | Yes (1) or No (0) | Already binary variable |
| Parkinson’s disease (self-reported) | Yes (1) or No (0) | Already binary variable |
| Dementia (self-reported) | Yes (1) or No (0) | Already binary variable |
| Arthritis (self-reported) | Yes (1) or No (0) | Already binary variable |
| Any other chronic disease (self-reported) | Yes (1) or No (0) | Already binary variable |
| Polypharmacy (self-reported) | >4 medications (1), ≤4 medications (0) | Previous literature (Theou et al. 2013) |
| Body Mass Index (BMI) | 18.5 to <25 (0), 25 to <30 (0.5), <18.5 or >equal to 30 (1) | Previous literature (Chamberlain et al. 2016) |
| 6m walk time (gait speed) | >10 seconds or physically unable (1), <10 seconds (0) | Previous literature (Hoogendijk et al. 2017) |
| Able to stand up from a chair | Yes (1) or No (0) | Already binary variable |
| Grip strength (strongest hand and stratified by sex and BMI) | Bottom 5^th^ percentile (1), 5^th^-20^th^ percentile (0.5), Above 20^th^ percentile (0) | Recommended technique where no established cut-offs available (Theou et al. 2015) |
| Townsend Disability Scale (Townsend 1979) | 11 – 18 (1), 0 -10 (0) | Previous literature (Matthews et al. 2016) |
| Peak Expiratory Flow rate (stratified by sex) | Bottom 5^th^ percentile (1), 5^th^-20^th^ percentile (0.5), Above 20^th^ percentile (0) | Recommended technique where no established cut-offs available (Theou et al. 2015) |
| Forced expiratory volume (stratified by sex) | Bottom 5^th^ percentile (1), 5^th^-20^th^ percentile (0.5), Above 20^th^ percentile (0) | Recommended technique where no established cut-offs available (Theou et al. 2015) |
| Depression (measured by the HADS)(Zigmond and Snaith 1983) | 11 -21 (1), 8 – 10 (0.5), 0 – 7 (0) | Previous literature (Zigmond and Snaith 1983) |
| Anxiety (measured by the HADS)(Zigmond and Snaith 1983) | 11 -21 (1), 8 – 10 (0.5), 0 – 7 (0) | Previous literature (Zigmond and Snaith 1983) |
| Mini-Mental State Examination (MMSE)(Folstein, Folstein, and McHugh 1975) | <10 (1), 11-17 (0.75), 18 – 20 (0.5), 20 – 24 (0.25), >24 (0) | Previous literature (Searle et al. 2008) |
| Digit Symbol(Wechsler 2003) | Bottom 5^th^ percentile (1), 5^th^-20^th^ percentile (0.5), Above 20^th^ percentile (0) | Recommended technique where no established cut-offs available (Theou et al. 2015) |
| Block Design(Wechsler 2003) | Bottom 5^th^ percentile (1), 5^th^-20^th^ percentile (0.5), Above 20^th^ percentile (0) | Recommended technique where no established cut-offs available (Theou et al. 2015) |
| Verbal Fluency(Wechsler 2003) | Bottom 5^th^ percentile (1), 5^th^-20^th^ percentile (0.5), Above 20^th^ percentile (0) | Recommended technique where no established cut-offs available (Theou et al. 2015) |
| Matrix Reasoning(Wechsler 2003) | Bottom 5^th^ percentile (1), 5^th^-20^th^ percentile (0.5), Above 20^th^ percentile (0) | Recommended technique where no established cut-offs available (Theou et al. 2015) |
| Reaction time test(Cox, Huppert, and Whichelow 1993) | Bottom 5^th^ percentile (1), 5^th^-20^th^ percentile (0.5), Above 20^th^ percentile (0) | Recommended technique where no established cut-offs available (Theou et al. 2015) |
| Delayed recall(Wechsler 2003) | Bottom 5^th^ percentile (1), 5^th^-20^th^ percentile (0.5), Above 20^th^ percentile (0) | Recommended technique where no established cut-offs available (Theou et al. 2015) |

*Note*. HADS: Hospital Anxiety and Depression scale

**Online Resource 2: Deriving the Fried Criteria**

The Fried Criteria was comprised on five dimensions. These were measured in the LBC1936 as follows;

**Weight loss;** Weight was measured using an electronic weighing scale, and height was measured in metres using a stadiometer. From this, it was possible to compute BMI by dividing weight by height squared. At baseline, weight loss was defined as a BMI less than 18.5 kg/m^2^. At waves 3 and 4, weight loss was defined as a loss of weight of 10% or more since their previous visit or a BMI less than 18.5 kg/m^2^.

**Exhaustion;**

Exhaustion was measured using the Hospital Anxiety and Depression Scale (HADS) (Zigmond and Snaith 1983). Exhaustion was scored as present if the participant responded ‘very often’ or ‘nearly all the time’ to the item ‘I feel as if I’m slowed down’.

**Physical activity;**

A question asking participants about their usual level of physical activity was used with six responses ranging from moving only when necessary, to heavy exercise or sport several times a week. In line with previous publications (Gale et al. 2017), participants in the lowest sex-specific 20% of the distribution were defined as having low physical activity.

**Walking speed;**

Participants were recorded walking a distance of six metres at maximum speed. After adjusting for sex and height, those in the lowest 20% of the distribution were considered to have a slow walking speed.

**Weakness;**

Maximum grip strength was measured in all participants using a dynamometer. Participants were measured three times with the strongest attempt being used for analysis. After adjusting for sex and BMI, those in the lowest 20% of the distribution were defined as having weakness.

**Online Resource 3: Defining occupational social class**

Occupational social class was based upon principal occupation, coded in line with the 1980 census (General 1991). Five social class categories were used: professional, managerial, skilled non-manual, skilled manual, and semiskilled/unskilled. The women in the cohort were asked for their husband’s occupation as well as their own, and they were assigned a social class based on the highest occupation of the household. This was derived from their own occupation for about half of the women, and from their husband’s occupation for the remainder.

Childhood IQ was derived from Moray House Test scores at age 11 (Penrose 1949). Raw scores were corrected for age in days at time of testing and converted to an IQ scale where mean (SD) = 100 (15).

**References**

Chamberlain, Alanna M, Jennifer L St Sauver, Debra J Jacobson, Sheila M Manemann, Chun Fan, Véronique L Roger, Barbara P Yawn, and Lila J Finney Rutten. 2016. 'Social and behavioural factors associated with frailty trajectories in a population-based cohort of older adults', *BMJ open*, 6: e011410.

Cox, Brian D, Felicia A Huppert, and Margaret J Whichelow. 1993. *The Health and Lifestyle Survey: Seven Years on: a Longitudinal Study of a Nationwide Sample, Measuring Changes in Physical and Mental Health, Attitudes and Lifestyle* (Dartmouth Publishing Group).

Folstein, Marshal F, Susan E Folstein, and Paul R McHugh. 1975. '“Mini-mental state”: a practical method for grading the cognitive state of patients for the clinician', *Journal of psychiatric research*, 12: 189-98.

Gale, Catharine R., Stuart J. Ritchie, Cyrus Cooper, John M. Starr, and Ian J. Deary. 2017. 'Cognitive Ability in Late Life and Onset of Physical Frailty: The Lothian Birth Cohort 1936', *Journal of the American Geriatrics Society*, 65: 1289-95.

General, Registrar. 1991. "Office of population censuses and surveys. Classification of occupations." In.: London: HMSO.

Hoogendijk, Emiel, Olga Theou, Kenneth Rockwood, Bregje Onwuteaka-Philipsen, Dorly Deeg, and Martijn Huisman. 2017. 'Development and validation of a frailty index in the Longitudinal Aging Study Amsterdam', *Aging Clinical & Experimental Research*, 29: 927-33.

Matthews, Fiona Elaine, BCM Stephan, L Robinson, C Jagger, Linda Elizabeth Barnes, A Arthur, C Brayne, Ageing Studies CFAS Collaboration, A Comas-Herrera, and R Wittenberg. 2016. 'A two decade dementia incidence comparison from the Cognitive Function and Ageing Studies I and II', *Nature communications*, 7: 11398.

Penrose, LS. 1949. 'The Trend of Scottish Intelligence: A Comparison of the 1947 and 1932 Surveys of the Intelligence of Eleven‐year‐old Pupils. Scottish Council for Research in Education. Univ. of London Press Ltd. 1949. Pp. 151+ xxviii. Price 7s. 6d', *Annals of Eugenics*, 15: 186-87.

Searle, Samuel D, Arnold Mitnitski, Evelyne A Gahbauer, Thomas M Gill, and Kenneth Rockwood. 2008. 'A standard procedure for creating a frailty index', *BMC geriatrics*, 8: 24.

Theou, O, MDL O ‘Connell, BL King-Kallimanis, AM O'Halloran, K Rockwood, and RA Kenny. 2015. 'Measuring frailty using self-report and test-based health measures', *Age and ageing*, 44: 471-77.

Theou, Olga, Thomas D Brothers, Arnold Mitnitski, and Kenneth Rockwood. 2013. 'Operationalization of frailty using eight commonly used scales and comparison of their ability to predict all‐cause mortality', *Journal of the American Geriatrics Society*, 61: 1537-51.

Townsend, Peter. 1979. *Poverty in the United Kingdom: a survey of household resources and standards of living* (Univ of California Press).

Wechsler, David. 2003. *WISC-IV: Administration and scoring manual* (Psychological Corporation).

Zigmond, Anthony S, and R Philip Snaith. 1983. 'The hospital anxiety and depression scale', *Acta Psychiatrica Scandinavica*, 67: 361-70.
